# Continuous Aerosolized Albuterol Treatment for Status Asthmaticus on the General Care Floor: A Quality Improvement Initiative

**DOI:** 10.1097/pq9.0000000000000896

**Published:** 2026-07-20

**Authors:** Kailey A. Remien, Charles Hardy, Karen Allen, Matthew Wysong, Morgan Zlotolow, Gerd McGwire, Courtney Whitacre, Renae Forsythe, Tara Dinh, Lisa Ulrich, Adjoa A. Andoh, Samantha W. Gee, Shauna Schord

**Affiliations:** From the * Division of Critical Care Medicine, Nationwide Children’s Hospital, Columbus, Ohio; † Center for Clinical Excellence, Nationwide Children’s Hospital, Columbus, Ohio; ‡ Division of Hospital Medicine, Nationwide Children’s Hospital, Columbus, Ohio; § Department of Pediatrics, The Ohio State University College of Medicine, Columbus, Ohio; ¶ Division of Pulmonary, Nationwide Children’s Hospital, Columbus, Ohio; ‖ Department of Respiratory Therapy, Nationwide Children’s Hospital, Columbus, Ohio; ** Division of Emergency Medicine, Nationwide Children’s Hospital, Columbus, Ohio.

## Abstract

**Introduction:**

Asthma, a leading cause of pediatric hospitalization, affects more than 6 million US children and costs more than $5 billion annually. Continuous aerosolized albuterol (CAA) effectively treats severe exacerbations but is typically reserved for intensive care or emergency settings. This quality improvement project aimed to safely expand CAA use on the general care floor (GCF) by monitoring treatment uptake, protocol adherence, and safety outcomes.

**Methods:**

This single-center quality improvement project, led by a multidisciplinary team, included children aged 2–18 years with status asthmaticus on CAA who met the inclusion criteria. Interventions involved protocol development, staff education, electronic health record updates, and a resident-led handoff huddle. Primary outcomes were non–pediatric intensive care unit (PICU) CAA hours and the proportion of patients treated on the GCF. Process measures included protocol use and handoff huddles. Balancing metrics included PICU and emergency transfers, fluid-refractory hypotension, and ED length of stay.

**Results:**

From June 2024 to October 2025, GCF CAA accounted for 17% of total hospital CAA, surpassing the 10% target. ED length of stay remained unchanged, indicating no disruption to workflow. We safely treated 72 patients on the GCF (average CAA duration of 21.6 h); 22 (31%) required PICU transfer; none were emergent transfer. This intervention saved 1,017 PICU hours (42.4 PICU bed-days), equivalent to $275,600 in room-and-board costs. These savings represent a substantial reduction in PICU resource use; however, we did not conduct a formal cost-effectiveness analysis.

**Conclusions:**

CAA can be safely administered in the GCF following a structured protocol, reducing the need for PICU resources without adversely affecting ED care efficiency.

## INTRODUCTION

Asthma is the third leading cause of pediatric hospitalization.^1,2^ Continuous aerosolized albuterol (CAA) is commonly used to treat severe asthma exacerbations.^3–7^ CAA is primarily used in emergency departments (EDs) and pediatric intensive care units (PICUs).^7,8^ CAA use in PICUs is associated with higher costs and longer hospital stays than CAA use on general care floors (GCFs).^5,8–10^ Some children’s hospitals use CAA on the GCF, finding it safe and at least as effective as intermittent dosing for asthma treatment.^7,10–12^ A large inpatient retrospective cohort study found that delivering CAA in a non–intensive care unit (ICU) setting under a standardized protocol resulted in low rates of clinical deterioration and few adverse events.^7,10^ However, published descriptions often lack operational details, limiting reproducibility.

This quality improvement (QI) project aimed to safely initiate CAA on the GCF, where hospital policy previously prohibited it. Due to this restriction, our institution experienced PICU bed shortages and ED boarding, leading to this QI project. Our specific aim was to safely initiate CAA use on the GCF for at least 10% of patients requiring CAA treatment from June 2024 to October 2025. We selected 10% as a safety-focused initial target, allowing for measurable impact while closely monitoring balancing measures to mitigate unintended harm. Outcomes, process measures, and balancing measures were tracked.

## METHODS

We conducted this QI initiative at a large academic children’s hospital. During the 5-year study period, the GCF averaged 629 asthma admissions annually, and the PICU averaged 238. The GCF is a general ward without a step-down unit, with nurse-to-patient ratios of 1:3 to 1:5, compared with 1:1 to 1:2 in the PICU. The care team checks vital signs at least every 4 hours; patients have access to continuous pulse oximetry and cardiac monitoring (not telemetry), with rapid escalation available through the rapid response team. Our rapid response team consists of the primary team, the safety officer of the day (SOD), and a PICU representative (typically a fellow).

**Fig. 1.**
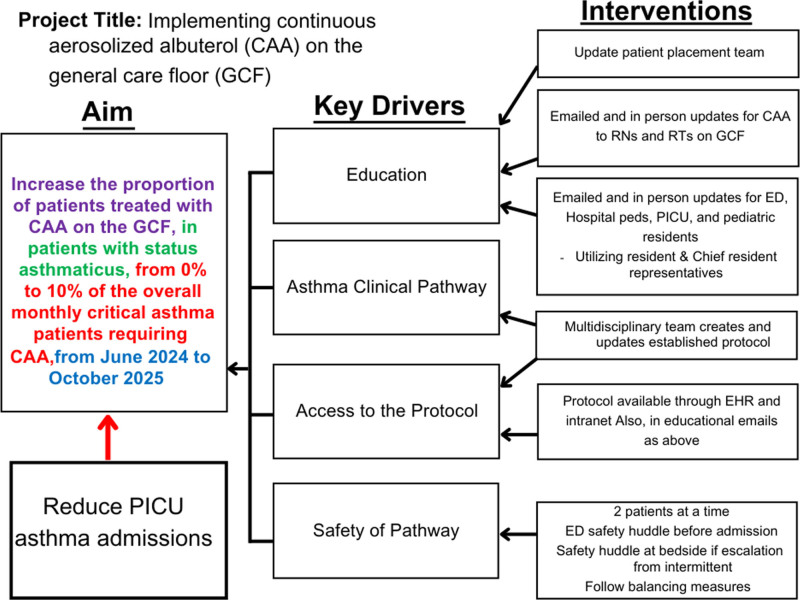
Key driver diagram.

A multidisciplinary group, including physicians from hospital pediatrics, emergency medicine, PICU, and pulmonary medicine; pediatric residents; respiratory therapists (RTs); nursing (RN) leadership; and a quality specialist, met regularly to develop this initiative. The QI team did not include families or patients, nor did it gather their perspectives. This multidisciplinary group created a key driver diagram (Fig. 1) using tenets of the Model for Improvement.^13^

**Fig. 2.**
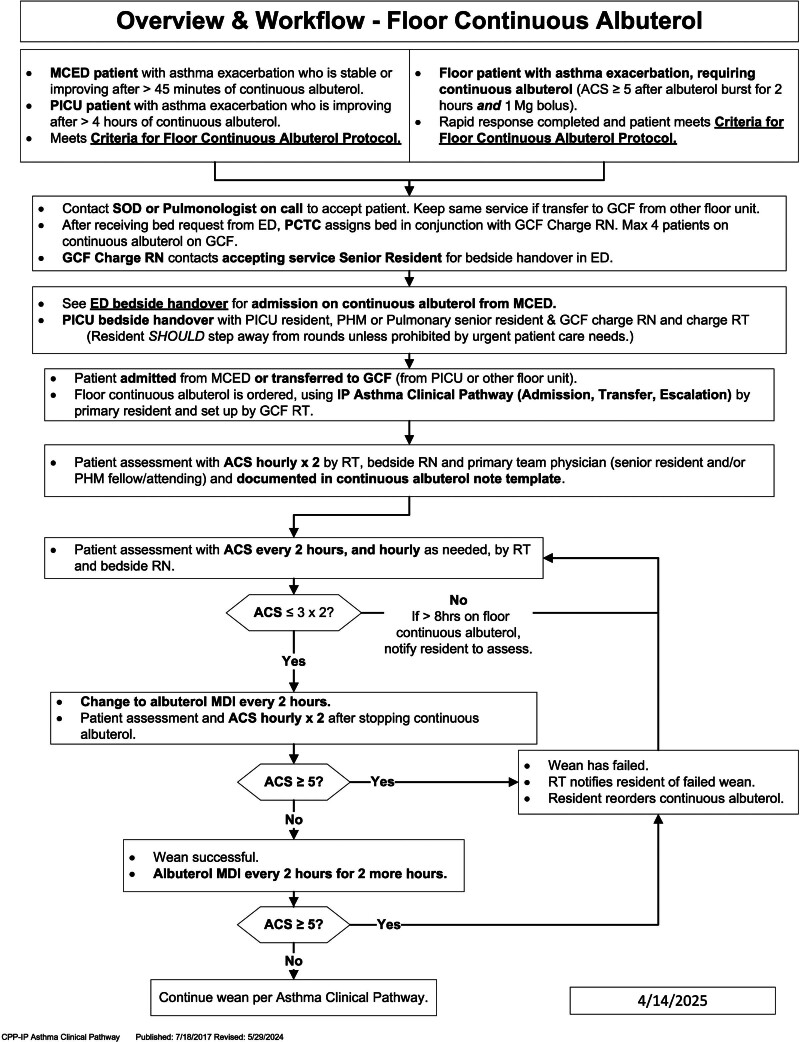
Asthma clinical pathway: overview and workflow for floor continuous albuterol. Criteria for continuous albuterol protocol links to Supplemental Figure 1 (**Supplemental Digital Content 2**, https://links.lww.com/PQ9/A777). PCTC, physician consult/transfer center.

Our institution uses a standardized inpatient asthma pathway that incorporates the Asthma Clinical Score (ACS) to assess severity and guide bronchodilator escalation and weaning. (**See figure 2, Supplemental Digital Content 1**, which displays the ACS rubric. SaO_2_, saturation of oxygen; tachypnea, rapid breathing, https://links.lww.com/PQ9/A778.) CAA on the GCF was built into this pathway. Albuterol titration and weaning are ACS-driven and led by RTs in conjunction with the treating providers. We aligned the electronic health record (EHR) and pathway additions with the QI rollout to support reliable adoption, and this has also been shown to reduce length of stay (LOS).^14^ Other asthma QI projects attribute practice-change success to order set implementation.^9^ We continued bedside staff-led (RT, RN, or physician), ACS-guided weaning in the CAA protocol, an approach associated with shorter LOS without worsening outcomes.^8,14,15^

## INCLUSION/EXCLUSION CRITERIA

Eligible patients included children aged 2–18 years with an acute asthma exacerbation requiring CAA. This age range was selected to minimize overlap with bronchiolitis, to align with commonly used asthma pathway criteria, and to exclude adults. The diagnosis of an acute asthma exacerbation was made by the clinician caring for the patient. Patients did not require a previous diagnosis of asthma. Asthma admissions were defined as anyone with an ICD-10 code for asthma (J45%) on the hospital problem list or as a primary or secondary billing diagnosis. All patients treated with CAA on the GCF during the study were part of the analysis.

We excluded patients who received CAA in the PICU or ED but not on the GCF, those with significant comorbidities, severe respiratory failure or hypoxia, hemodynamic instability, concern for bacterial pneumonia, or those who required critical medications for status asthmaticus. Details are shown in Supplemental Figure 1. (**See figure 1, Supplemental Digital Content 2**, which displays the inclusion and exclusion criteria for CAA on the GCF. FiO_2_, fraction of inspired oxygen; PPV, positive-pressure ventilation, https://links.lww.com/PQ9/A777.) Patients treated at outside EDs or other hospitals were not eligible.

### Interventions

#### Updating the Asthma Clinical Pathway

Before this initiative, the institutional asthma pathway did not allow for CAA on the GCF. The team revised the pathway to include CAA on the GCF for patients who met the inclusion criteria. Updates were made to the EHR (Epic Systems, Verona, WI) asthma order set to include the new CAA pathway. Our institution has a large pediatric ED on the main hospital campus and a stand-alone pediatric ED off campus. Only the main campus ED (MCED) participated in this initiative due to handoff huddle requirements.

#### Instituting an ED Huddle for Handoff

Before admitting patients to the GCF on CAA, the GCF team evaluates them during a huddle. This is a brief, interdisciplinary communication that occurs before a patient is transferred from the MCED to the GCF on CAA. The huddle is initiated by the receiving unit charge nurse. The huddle confirms eligibility for GCF CAA and that everyone is comfortable with the patient’s clinical picture. Huddle completion is documented using a standardized EHR template. Huddles in the ED before admission are common in our MCED. They have been well received by floor staff, and complaints of an increased workload have been rare. Data were not obtained on staff perceptions of the huddle as it pertains to CAA on the GCF.

**Fig. 3.**
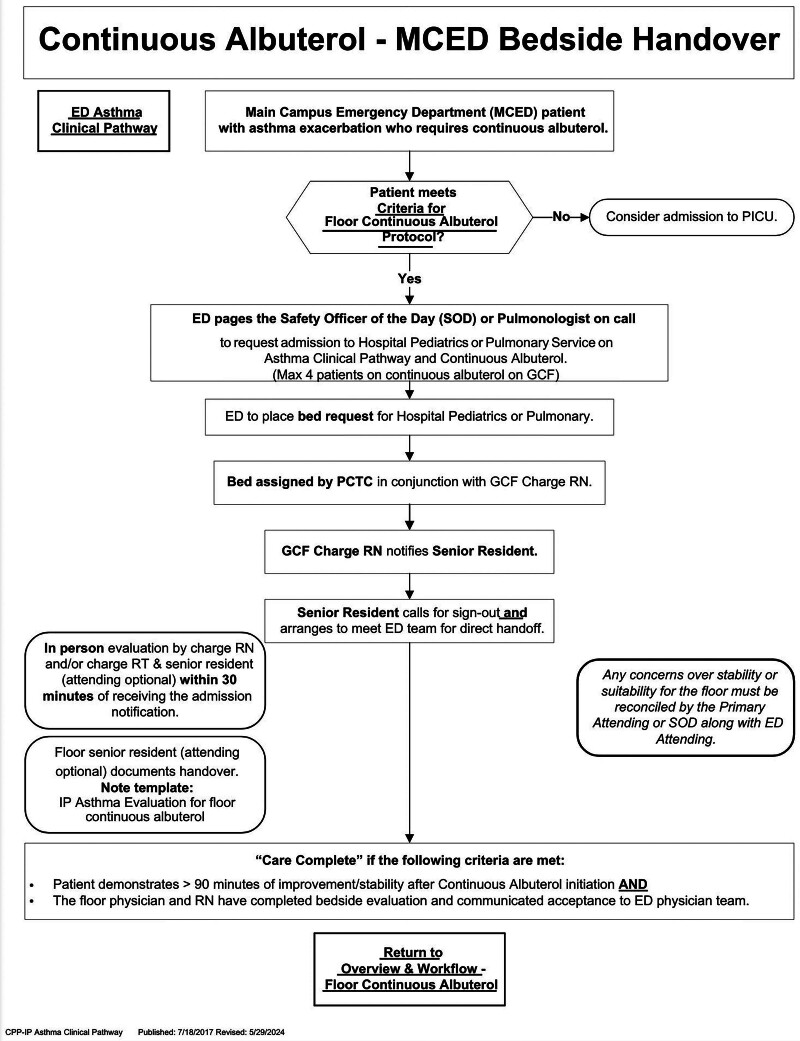
Asthma clinical pathway workflow for ED-to-floor admissions for CAA and the GCFCAA protocol. IP, inpatient; PCTC, physician consult/transfer center.

To begin the process of being accepted for CAA on the GCF, patients with status asthmaticus must remain stable on CAA in the ED for at least 45 minutes, with stability defined as no worsening or improvement in the ACS (**Supplemental Digital Content 1,** https://links.lww.com/PQ9/A778). Once patients meet these criteria, the ED team discusses the patient with the SOD, an on-call pediatric hospital medicine (PHM) attending who approves PHM admissions. The SOD serves as backup if the PHM senior resident is unavailable for the huddle, acts as the escalation point for safety concerns, and is part of the rapid response team. The workflow for admitting a patient to the GCF on CAA and the treatment protocol are illustrated in Figures 2 and 3, respectively.

Once the ED attending or fellow discusses the patient with the admitting attending, the patient placement team assigns a room on the GCF. The GCF charge RN contacts the accepting service’s senior resident by paging or secure EHR chat to coordinate the in-person huddle. Residents lead the handoff huddle, with the accepting senior resident acting as the primary physician representative. This structure emphasizes resident autonomy while ensuring oversight; other team members, such as the intern or charge RT, may participate in the huddle. The minimum personnel required for the handoff huddle are the receiving charge RN and the senior resident. If the accepting service’s senior resident and the charge RN agree that the patient is suitable for admission to the floor, the patient can be admitted after 90 minutes of stability on CAA in the ED. If there are *any* concerns about eligibility or safety, the patient is admitted to the PICU. Most concerns were based on clinical gestalt or high ACS scores, despite stability. Patients were initially cohorted on 1 unit for closer monitoring.

#### Alternate Routes to CAA on the GCF

Patients on intermittent aerosolized albuterol may escalate to CAA, requiring a rapid response huddle on the GCF instead of an ED huddle. The rapid response huddle involves discussion and patient evaluation by the primary treatment and rapid response team members. If patients are deemed safe and eligible for CAA on the floor, they are transferred to the appropriate unit and service. Patients with CAA who do not require intravenous bronchodilators or positive-pressure ventilation can also be transferred from the PICU to the GCF.

#### Education

Before the first plan-do-study-act (PDSA) cycle, we educated RTs, RNs, residents, and physicians from the ED, PICU, and PHM services regarding the admission process and treatment protocol. We delivered education during each group’s regularly scheduled, established meetings. We shared asthma algorithms via email, the hospital intranet, and as links on the ED and admission order sets within the EHR. The patient placement team also received training on the updated admission matrix. The patient placement team comprises nurses who assign the floor and bed placement as well as connect triage calls from outside hospitals and physicians.

### PDSA Cycle 1

We launched the protocol on a single unit with designated RT staffing. To prioritize feasibility and safety, we limited the unit to 2 concurrent CAA patients and restricted eligibility to the PHM service. Patients on GCF CAA were monitored every 2 hours, more frequently than typical asthma patients, reflecting the higher staffing demands of this care model.

### PDSA Cycle 2

After implementing GCF CAA delivery, we expanded capacity to 4 patients at once and broadened eligibility to include the inpatient pulmonary service. The ED alternates CAA admissions between pulmonary and PHM unless the patient has an established pulmonology relationship. This addressed observed patient and family distress when pulmonary patients required escalation and transfer by expanding GCF CAA to the pulmonary service.

### Study of the Interventions

#### Measures

**Fig. 4.**
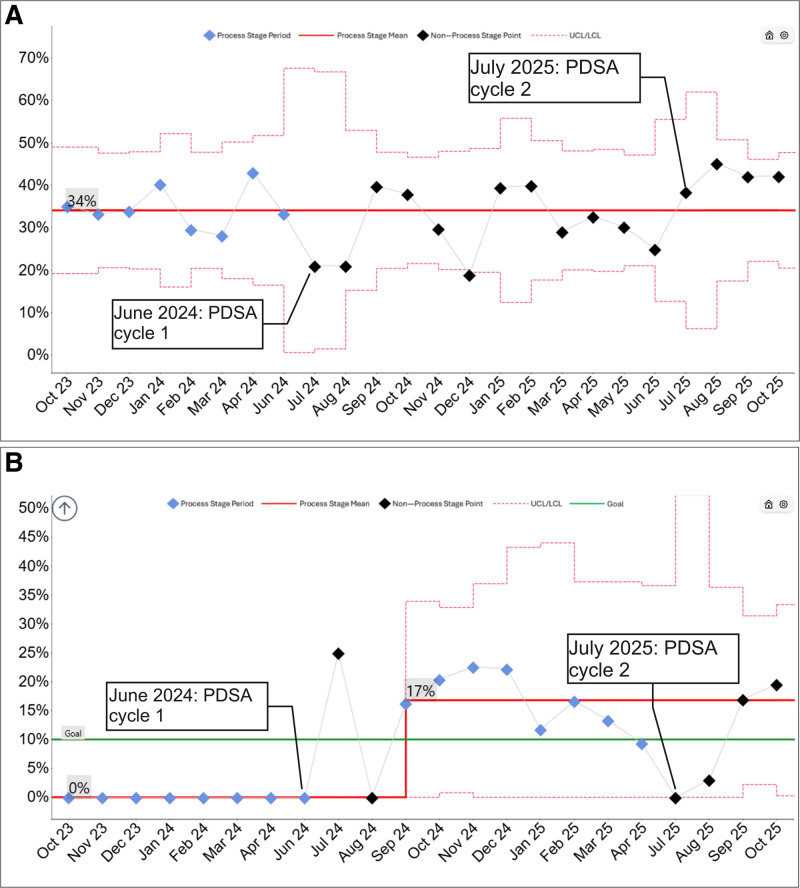
Continuous aerosolized albuterol utilization over time since initiation. A, P-chart illustrating the percentage of patients with status asthmaticus receiving CAA relative to the total number of patients with status asthmaticus over time. B, P-chart illustrating the percentage of patients with status asthmaticus receiving CAA on the floor relative to the total number of patients with status asthmaticus receiving CAA over time. LCL, lower control limit; UCL, upper control limit.

We selected measures to assess effectiveness (outcomes), implementation reliability (process), and unintended consequences (balancing). Outcome measures included the number of non-PICU CAA hours, the proportion of asthma patients receiving CAA on the GCF relative to all patients receiving CAA, and PICU hours saved. We estimated PICU hours saved by non-PICU albuterol use because these patients had previously received treatment in the ICU. Process measures included the use of the asthma pathway order set and completion of standardized handoff huddle documentation. Balancing measures included ED LOS to ensure that our protocol did not prolong ED stay or increase PICU transfers or emergency transfers^13^ (defined as requiring intubation, significant fluid resuscitation, or vasoactive medications within an hour of ICU transfer) or episodes of fluid-refractory hypotension. Fluid-refractory hypotension was defined as requiring 60 mL/kg or more of fluid resuscitation or a total fluid volume of 3 L or more.^16^ We monitored fluid-refractory hypotension because CAA can cause diastolic hypotension in children.^15^ We also compared CAA use with that of the previous year to verify that increases were not solely due to treatment availability (Fig. 4A). Although PICU bed shortages and ED boarding contributed to the local rationale for this initiative, these factors were not prospectively quantified as part of the project.

We emphasized clear communication and ongoing feedback between frontline staff and the improvement team. Bedside staff escalated concerns to the charge RN or RT, who then engaged the improvement team. Stakeholders met regularly to address issues and provide real-time email support, ensuring feedback-informed adjustments that guided interventions during team discussions.

### Analysis

This project used statistical process control (SPC) methods to monitor our primary outcome measures. We generated SPC charts with Easy SPC (BCN Group Ltd., 2025). We analyzed demographic data using *P* values calculated with 2-part testing or 1-way analysis of variance, as appropriate, in QI Macros (KnowWare International, Denver, CO) or a Microsoft Excel 365 add-in (version 2410; Microsoft Corporation, Seattle, WA).

### PICU Transfers

Patients who initiated CAA on the GCF and required transfer to the PICU remained classified in the GCF group, because some of their therapy occurred outside the PICU. We assigned CAA hours based on their location at the time of treatment: GCF hours before transfer and PICU hours afterward.

### Ethical Considerations

This study was classified as nonhuman research by our hospital’s institutional review board.

### Considerations of Race and Ethnicity

We included race as a social construct that may reflect differential access to care or structural racism. Notably, status asthmaticus exhibits racial differences in severity.^17^ It was not considered a proxy for genetic or biological differences. We included racial data to analyze potential disparities in our population. The EHR primarily supplied racial data through patient or caregiver self-identification at hospital registration.

## RESULTS

Between June 2024 and October 2025, 72 patients received CAA on the GCF out of 467 total CAA patients. The average age was 9.04 years. The population was 39% male and 34% African American or Black, consistent with US asthma data.^17^ Other patient information can be seen in Supplemental Table 1. (**See table 1, Supplemental Digital Content 3**, which displays a demographic table, https://links.lww.com/PQ9/A780.)

**Fig. 5.**
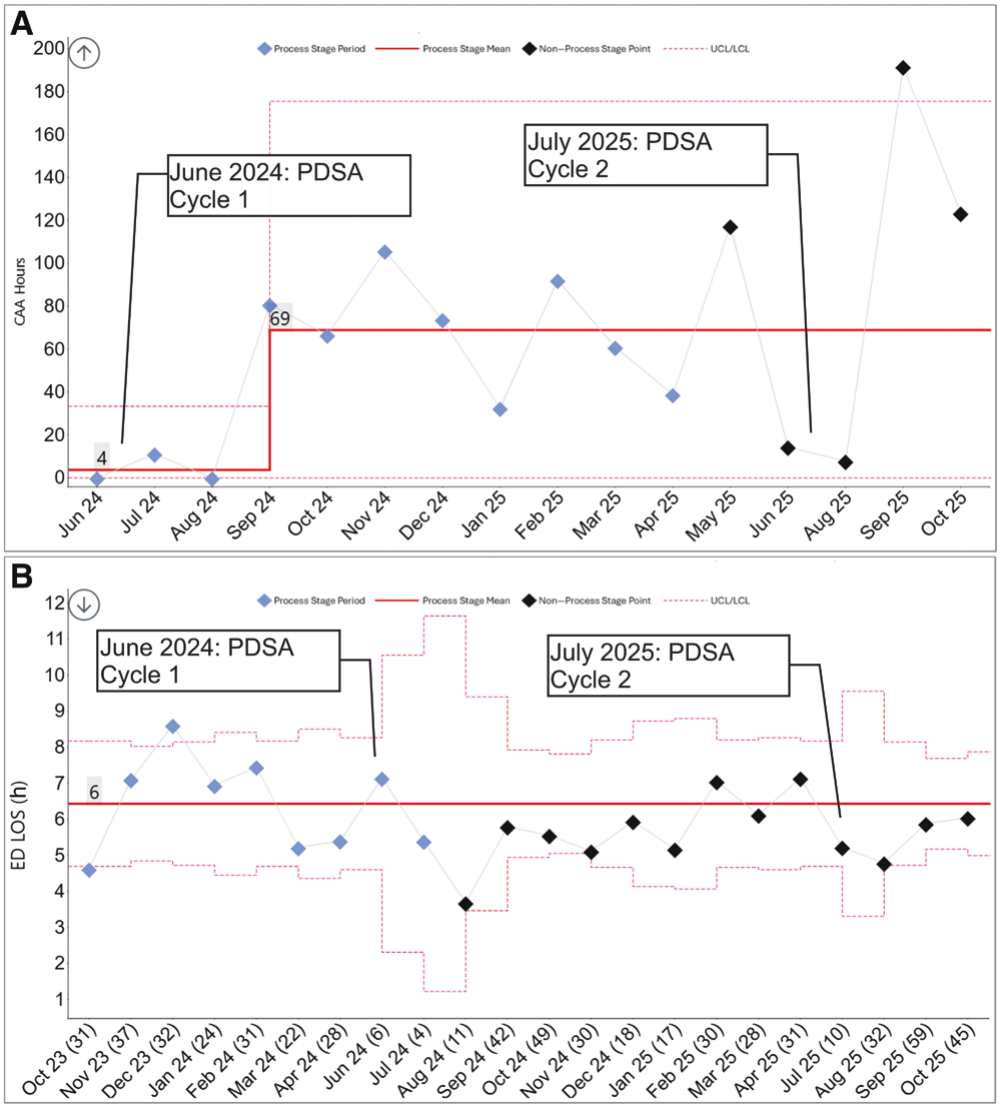
CAA hours on the GCF over time showing improvement in resource utilization and ED LOS (balancing measure) over time showing no worsening in LOS since implementation. A, I-chart displaying the ICU hours saved through CAA on the general care floor over time. B, X-bar chart showing the average LOS in the ED for all patients with status asthmaticus requiring CAA. LCL, lower control limit; UCL, upper control limit.

Because the GCF initially did not allow CAA, clinicians did not treat any acute asthma exacerbations with CAA outside the PICU or ED. On average, the team treated 17% of patients who needed CAA outside the PICU, surpassing our 10% goal (Fig. 4B). The I-chart shows an average of 69 CAA hours per month on the GCF (ICU hours saved) (Fig. 5A). Our 72 patients received a total of 1554 hours of CAA, with 1017 hours on the GCF. The length of CAA administration data is shown in Supplemental Table 1 (**Supplemental Digital Content 3,** https://links.lww.com/PQ9/A780).

Since this protocol began, the average monthly number of CAA treatments has remained steady (Fig. 4A), indicating that initiating CAA on the GCF did not increase usage. We based cost savings estimates on the difference in hospital room-and-board charges between the PICU ($12,300) and GCF ($5,800),^18^ serving as an estimate. Charges drop daily at midnight. We saved 1017 PICU hours, 42.4 PICU bed-days, and $275,600 in possible cost savings, while reducing PICU resource use.

### Process Measures

Process measures included using protocol order sets and huddle documentation with a standardized template. (**See figure 3, Supplemental Digital Content 4**, which displays the P-chart showing the percentage of patients receiving CAA on the general care floor who had a handoff huddle documented using the standardized template. The template also includes standardized dropdown boxes for easier documentation. 2025 Epic Systems Corporation. H8A, the initial unit in our hospital where CAA is allowed, https://links.lww.com/PQ9/A779.) Providers placed orders using the CAA albuterol order set in 95% of cases, promoting standardized asthma care. Clinicians documented only 56% of huddles using the template.

### Balancing Measures

Before this initiative, CAA patients had an average ED LOS of 6 hours (**Supplemental Digital Content 3**, https://links.lww.com/PQ9/A780). Since implementing the protocol, the ED LOS center line has remained steady (Fig. 5B), indicating no increase in ED LOS. Those who ultimately required PICU transfer spent an average of 6 hours in the ED, whereas those treated only on the GCF spent 7.1 hours; this difference was not statistically significant (*P* = 0.07). A total of 8 patients escalated to CAA after intermittent albuterol, and 22 patients required PICU transfer after CAA on the GCF. None of these patients met emergency transfer criteria. No patients transferred out of the PICU on CAA.

Thirteen patients received a fluid bolus. Only 6 were due to hypotension, characterized by changes in vital signs rather than clinical deterioration. Seven patients received fluid prophylactically with a magnesium bolus. No patients required more than 1 fluid bolus (20 mL/kg or 1 L) for hypotension, indicating none had fluid-refractory hypotension.

### Incorporating Feedback

After the first PDSA cycle, frontline RT and RN staff reported that the process worked well. The admission process and huddles were feasible but required clearer guidance on how to notify the resident team. The charge RN was then made responsible for initiating contact for the ED huddle. RN and RT leaders observed that pulmonary patients who needed escalation to CAA and transfer to PHM experienced patient/family distress due to existing relationships. This observation led to the expansion of GCF CAA to the pulmonary service in the second PDSA cycle. Successes included high adherence to protocols and increased resident confidence in leading huddles. Challenges involved delays in senior resident arrival within 30 minutes, prompting workflow adjustments and backup coverage by the SOD. These refinements, driven by frontline feedback, support the QI framework.

## DISCUSSION

This initiative suggests that, within our institution’s implementation conditions (limited concurrent patient volume, structured RT-driven monitoring, and ED preadmission huddles), clinicians can administer CAA on the GCF for pediatric patients with status asthmaticus, thereby supporting broader access outside the ED and PICU. Although prior studies confirm safety, they often lack detailed implementation strategies, such as PDSA testing and frontline feedback. Our protocol is the first to require resident-led handover huddles and provides a practical, reproducible framework. Our QI initiative has preserved healthcare resources by managing eligible CAA patients on the GCF, avoiding PICU stays, and reducing room-and-board charges. Although these patients often meet critical care billing criteria, avoiding PICU admission results in cost savings and optimal use of intensive care services.^18^

Patient safety remained the top priority throughout this initiative. We administered CAA on the GCF in accordance with specific clinical criteria to ensure proper patient selection. No patients required emergent PICU transfer (requiring intubation, significant fluids, or vasoactive medications within 1 h of ICU transfer), and none had fluid-refractory hypotension. Only 31% required transfer to the PICU. Any patients raising safety concerns during the handoff huddle were admitted to the PICU. The standardized asthma pathway and CAA protocol included clear escalation routes to the PICU for deterioration or at staff discretion. Additionally, education and structured communication with GCF staff and leadership supported informed decision-making and helped minimize variability in practice. We intentionally capped concurrent CAA use to 2 patients, then 4 patients on the GCF at a time as a built-in safeguard to maintain appropriate staffing, monitoring, and escalation capacity.

Our intervention featured novel huddles that facilitated efficient handoffs, interdisciplinary communication, and enhanced situational awareness. It could be resident-led without an attending physician, promoting resident autonomy in decision-making. This model is well suited for teaching institutions seeking to balance patient safety with medical education.

Future directions for this QI initiative include continuing to scale and refine the CAA process. The third PDSA cycle will expand CAA use to a second inpatient unit. Including the stand-alone ED will be a focus in the future to ensure all eligible patients are included. Using standardized handoff huddle templates can improve process reliability. Future PDSA cycles will aim to reduce ED LOS. In addition, future studies should incorporate formal cost analyses.

Beyond effectiveness, sustainability is also important when considering broader implementation of this approach. This intervention also includes features that may support sustainability, such as integration into the clinical pathway, a standardized workflow, and bedside staff-led, ACS-guided weaning. Continued education, adherence monitoring, and reinforcement of patient selection criteria will remain important to sustaining safe implementation over time.

This study has limitations. We conducted this project at a single pediatric center with a strong QI culture and a supported Clinical Pathways Program. Although safety outcomes were favorable, the study lacked the power to detect rare adverse events. The proportion treated on the GCF was limited because we capped the number of concurrent CAA patients; many were ineligible for the GCF and went to the PICU. PICU resource use improved, but we did not perform a formal cost-effectiveness analysis. We did not prospectively capture huddle decisions for patients not admitted to the GCF (and denials were not routinely documented), which limited our ability to characterize reasons for nonacceptance and to identify targeted changes to safely expand eligibility.

## CONCLUDING SUMMARY

This QI initiative provides a protocolized framework for CAA administration on the GCF. Other institutions can adapt this intervention to expand access to CAA. The initiative has improved PICU resource use, maintained safety, and established a scalable team communication strategy. These findings can inform future high-acuity asthma care in non-ICU settings and promote collaboration and resident leadership.

## ACKNOWLEDGMENTS

The authors thank the nurses, RTs, and physicians across multiple specialties and levels of training who contributed to this study through their collaboration and feedback.

## Supplementary Material


