# Supplementary material for: Continuous Aerosolized Albuterol Treatment for Status Asthmaticus on the General Care Floor: A Quality Improvement Initiative
Source: Pediatr Qual Saf. 2026 Jul 20;11(4):e896. doi: 10.1097/pq9.0000000000000896 (PMC13375096; doi:10.1097/pq9.0000000000000896)
Supplement: Supplementary file 1 [file pqs-11-e896-s001.pdf]

Asthma Clinical Score (ACS)

**Scoring Key** (Maximum score is "13")	**0**	**1**	**2**	**3**	**4**
**Tachypnea** *(see reference)*	No	Yes			
**O2 Requirement** to keep SaO2 ≥ 92%	RA	≤ 2 liters/31%	≤ 2 liters/31% ≤ 4 liters/ 50%	> 4 liters/50%	
**Wheezing**	None	End expiratory or scattered wheeze	Expiratory wheeze throughout	Inspiratory and expiratory wheeze	"Silent chest" (no air movement)
**Air Movement**	Normal/Good	Fair	Tight	Silent	
**Retractions** *(see reference)*	None	One type of retraction	Two or more types of retractions		

Asthma Exacerbation Severity

- Mild Respiratory distress (ACS ≤ 4)
- Moderate Respiratory distress (ACS 5 – 9)
- Severe Respiratory distress (ACS ≥ 10)
