# Supplementary material for: Continuous Aerosolized Albuterol Treatment for Status Asthmaticus on the General Care Floor: A Quality Improvement Initiative
Source: Pediatr Qual Saf. 2026 Jul 20;11(4):e896. doi: 10.1097/pq9.0000000000000896 (PMC13375096; doi:10.1097/pq9.0000000000000896)
Supplement: Supplementary file 2 [file pqs-11-e896-s002.pdf]

Continuous Albuterol Floor Protocol

Asthma Pathway - Continuous Albuterol Floor Protocol

Hospital Pediatrics and Pulmonary Service, GCF unit

Inclusion Criteria

- Patient must have primary condition of asthma exacerbation with no exclusion criteria as below
- Patient must be between 2 - 18 years of age
- No more than 4 patients on GCF on continuous albuterol. "Safety Officer of the Day" (SOD) or Pulmonologist on call, along with Respiratory Therapy, will ensure that no more than 4 patients are on continuous albuterol at any one time.

Indication/Usage

- **PICU transfer** of established, stable, and ideally weaning, asthma pathway patient on continuous albuterol for > 4 hours
- **Admission from MCED** of stable or improving asthma pathway patient on continuous albuterol for > 45 minutes and with asthma clinical score (ACS) stable or decreasing at time of bedside handover
- **Floor patient with asthma exacerbation** and continued respiratory distress, increased work of breathing and/or ACS ≥ 5 ***and*** one Mg bolus ***and*** approved by the acute care team.

Exclusion Criteria

- Age < 2 or > 18 yrs
- Patients in severe respiratory failure (PPV requirement, altered mental status, bradycardia, poor perfusion)
- FiO_2 requirement ≥ 50%
- MCED or PICU patient that received epinephrine, ketamine, continuous magnesium, terbutaline, or aminophylline
- History of recent intubation for current illness
- Known concurrent bacterial pneumonia
- Patients with significant comorbidities (cardiac, pulmonary, or neuromuscular disease, craniofacial abnormalities, immunodeficiency)
