# Supplementary material for: Continuous Aerosolized Albuterol Treatment for Status Asthmaticus on the General Care Floor: A Quality Improvement Initiative
Source: Pediatr Qual Saf. 2026 Jul 20;11(4):e896. doi: 10.1097/pq9.0000000000000896 (PMC13375096; doi:10.1097/pq9.0000000000000896)
Supplement: Supplementary file 3 [file pqs-11-e896-s003.pdf]

	All CAA (n=467)	ICU CAA (n=339)	GCF CAA (n=72)	GCF CAA with no PICU Admit (n=50)	GCF CAA with PICU Escalation (n=22)	p-value
Age (y)	7.56	7.41	9.04	8.52	10.23	0.25
**Sex**						
Male	282	206	39	28	11	0.64
Female	185	133	33	22	11	0.64
**Race**						
White	161	119	23	12	11	**0.034**
Black	224	160	34	24	10	0.84
Hispanic	31	21	7	6	1	0.24
Multi	37	28	6	6	0	**0.009**
Other	14	11	2	2	0	0.15
Non-English Speaking	33	24	6	5	1	0.37
**Insurance**						
Private	120	87	17	12	5	0.91
Public	307	225	48	33	15	0.86
Self-pay	29	21	6	4	2	0.88
Other	11	6	1	1	0	0.31
**Albuterol Hours**						
Total	12800.74	11584.69	1553.9	761.58	496.88	
Average	27.41	34.17	21.58	15.23	36.01	**0.00**
Total GCF	1016.77		1016.77	761.58	232.35	
Average GCF	14.12		14.12	15.23	10.56	**0.05**
Pre-PICU	232.35		232.35		232.35	
Average Pre-PICU	10.56		10.56		10.56	
PICU	10665.96	10665.95	537.13		537.13	
Average PICU	33.02	31.46	28.27		28.27	
**Length of Stay (Hrs)**						
ED	5.82	5.55	6.76	7.11	5.96	0.07
