# Supplementary material for: Continuous Aerosolized Albuterol Treatment for Status Asthmaticus on the General Care Floor: A Quality Improvement Initiative
Source: Pediatr Qual Saf. 2026 Jul 20;11(4):e896. doi: 10.1097/pq9.0000000000000896 (PMC13375096; doi:10.1097/pq9.0000000000000896)
Supplement: Supplementary file 4 [file pqs-11-e896-s004.pdf]

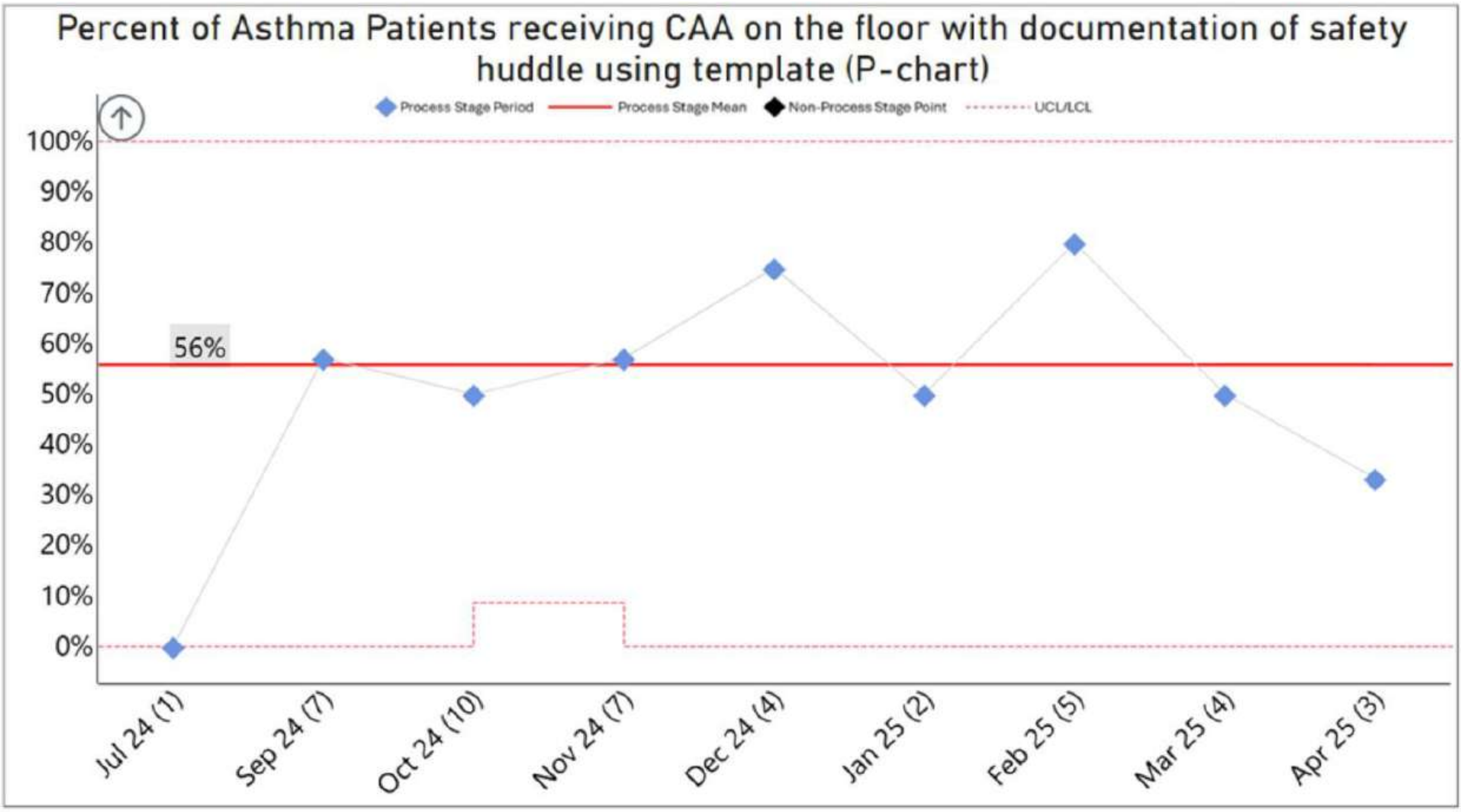

Evaluation for Floor Continuous Albuterol

Patient was evaluated for continuous albuterol on H8A.
Bedside evaluation +/- handoff: patient location
Team at bedside: IP Watcher Patient Plan

Asthma Clinical Score

None

Pertinent Exam Findings:
GENERAL: GENERAL EXAM: alert, well-appearing, no acut...
CARDIOVASCULAR: CARDIOVASCULAR EXAM: regular rate and rhythm, no mu...
CHEST: CHEST/LUNG EXAM: breath sounds clear and equal ...
ABDOMEN: ABDOMINAL EXAM: soft, nontender, nondistended,...
NEURO: NEUROLOGIC EXAM: alert, normal tone, no focal d...

Assessment: Maylond Gordon is a 6 year 1 month male with severity level asthma in status asthmaticus.

Contraindication(s) for floor continuous albuterol:
Contraindications for floor continuous albuterol

Plan:
Floor continuous albuterol plan
